# False Positive Results in SARS-CoV-2 Serological Tests for Samples From Patients With Chronic Inflammatory Diseases

**DOI:** 10.3389/fimmu.2021.666114

**Published:** 2021-05-03

**Authors:** Nastya Kharlamova, Nicky Dunn, Sahl K. Bedri, Svante Jerling, Malin Almgren, Francesca Faustini, Iva Gunnarsson, Johan Rönnelid, Rille Pullerits, Inger Gjertsson, Karin Lundberg, Anna Månberg, Elisa Pin, Peter Nilsson, Sophia Hober, Katharina Fink, Anna Fogdell-Hahn

**Affiliations:** ^1^ Department of Clinical Neuroscience, Karolinska Institutet, Stockholm, Sweden; ^2^ Center for Molecular Medicine, Karolinska Institutet, Stockholm, Sweden; ^3^ Department of Medicine Solna, Division of Rheumatology, Karolinska Institutet and Rheumatology, Karolinska University Hospital, Stockholm, Sweden; ^4^ Department of Immunology, Genetics and Pathology, Uppsala University, Uppsala, Sweden; ^5^ Department of Rheumatology and Inflammation Research, Institution of Medicine, Sahlgrenska Academy at University of Gothenburg, Gothenburg, Sweden; ^6^ Department of Clinical Immunology and Transfusion Medicine, Sahlgrenska University Hospital, Gothenburg, Sweden; ^7^ Division of Affinity Proteomics, Department of Protein Science, KTH Royal Institute of Technology, SciLifeLab, Stockholm, Sweden; ^8^ Department of Protein Science, KTH Royal Institute of Technology, Stockholm, Sweden; ^9^ Department of Neurology, Karolinska University Hospital, Stockholm, Sweden; ^10^ Centrum for Neurology, Academical Specialist Centrum, Stockholm, Sweden

**Keywords:** SARS-CoV-2, autoimmunity, autoantibodies, diagnostics, rheumatoid arthritis, systemic lupus erythematosus, multiple sclerosis, rheumatoid factor

## Abstract

Patients with chronic inflammatory diseases are often treated with immunosuppressants and therefore are of particular concern during the SARS-CoV-2 pandemic. Serological tests will improve our understanding of the infection and immunity in this population, unless they tests give false positive results. The aim of this study was to evaluate the specificity of SARS-Cov-2 serological assays using samples from patients with chronic inflammatory diseases collected prior to April 2019, thus defined as negative. Samples from patients with multiple sclerosis (MS, n=10), rheumatoid arthritis (RA, n=47) with or without rheumatoid factor (RF) and/or anti-cyclic citrullinated peptide antibodies (anti-CCP2) and systemic lupus erythematosus (SLE, n=10) with or without RF, were analyzed for SARS-CoV-2 antibodies using 17 commercially available lateral flow assays (LFA), two ELISA kits and one in-house developed IgG multiplex bead-based assay. Six LFA and the in-house validated IgG assay correctly produced negative results for all samples. However, the majority of assays (n=13), gave false positive signal for samples from patients with RA and SLE. This was most notable in samples from RF positive RA patients. No false positive samples were detected in any assay using samples from patients with MS. Poor specificity of commercial serological assays could possibly be, at least partly, due to interfering antibodies in samples from patients with chronic inflammatory diseases. For these patients, the risk of false positivity should be considered when interpreting results of the SARS-CoV-2 serological assays.

## Introduction

Severe acute respiratory syndrome coronavirus-2 (SARS-CoV-2) is the causative agent of the coronavirus disease 2019 (COVID-19), which emerged as a pandemic late 2019 (1). The cumulative number of infected and fatal cases can be followed at the Johns Hopkins University COVID-19 Dashboard (2). Patients with chronic inflammatory disease are often treated with immunomodulatory treatments and therefore potentially more susceptible to infections (3). As a result, there has been substantial concern during the pandemic as to the potential increased risk COVID-19 disease severity and mortality among these patient groups (4). There is limited evidence about their risk of severe COVID-19, or knowledge of how their disease or immunomodulatory treatment may affect either their pre-existing immunity or ability to develop protective immunity following infection (5, 6). Approximately 6% of the world’s population are affected by chronic inflammatory diseases which includes conditions such as multiple sclerosis (MS), rheumatoid arthritis (RA) and systemic lupus erythematosus (SLE) (7). These are generally progressive diseases and although for the majority there are no cures, treatment is centered around slowing disease progression with immunomodulatory treatments. The hallmarks of autoimmune diseases are inflammation, loss of self-tolerance and the presence of autoantibodies. MS is a chronic inflammatory disorder restricted to the central nervous system, characterized by demyelination, axonal loss and the formation of sclerotic plaques. The worldwide prevalence is estimated to be 2.2 million cases, but with large geographical variation (8). RA is a heterogeneous chronic inflammatory disease, which affected close to 5 million people globally by 2010 and with prevalence increasing due to the increased aging of the human population (9). The disease is characterized by synovial inflammation and the formation of the pannus, which causes cartilage and bone destruction, joint dysfunction, pain and disability. Rheumatoid factor (RF) and anti-citrullinated protein antibodies (ACPA), often detected as anti-cyclic citrullinated peptide (CCP) antibodies, are the most frequent and the most studied RA-related autoantibodies. RF is an antibody reactive with the Fc portion of IgG, mainly consisting of IgM in Caucasian RA populations, but also IgG and IgA RF are present. Although RF is detected in approximately 70% of RA patients, the presence of RF is not specific for RA. These autoantibodies are also present in a variety of other diseases as well as in the general population and may increase with age, smoking and chronic infection (10, 11). SLE is a systemic inflammatory disease of the connective tissue, characterized by a loss of self-tolerance and leading to production and deposition of a large panel of autoantibodies and immune complexes formation (12). Clinical manifestation of SLE is heterogeneous and can affect multiple organs. Approximately 25% of SLE patients have RF (13), but these patients can also have anti-nuclear antibodies (ANA) and anti- double-stranded (ds) DNA antibodies.

Serological tests are useful for determining past infection and present immunity. The presence of IgM antibodies indicates a recent infection, whereas presence of IgG antibodies indicates possible long-lasting immunity (14). Important information can be achieved by having access to reliable serological methods during a pandemic; to identify seropositive people for convalescent plasma donations; guide policies and ease restrictions on human mobility based on sero-epidemiological evidence; ensure immunity to allow key workers to return to work after exposure; and evaluate vaccine development studies and vaccine strategies.

Due to the substantial global demand, SARS-CoV-2 serological testing has been rapidly developed and released to the market. The assays are validated before release and also often independently verified before being approved (15, 16). However, the panel of samples used to determine specificity is often focused on ruling out cross-reactivity with other viral infections and might not include serum from patients with chronic inflammatory diseases, even though it is recommended (16). Based on experience from development and validation of serology assays for measuring anti-drug antibodies (ADA) in persons with chronic inflammatory disease, it is recommended to show specificity against drug naïve patient serum, as antibodies present in patients with autoimmune diseases are known to interact with reagents in serological assays and give unspecific signal (17, 18). Given the significant role serological tests may have as useful wide-spread screening tools for immunity, it is important to verify the specificity of SARS-CoV-2 serological tests in a similar way for specificity in patient groups with autoimmune diseases, using samples that were collected before the pandemic.

The aim of this pilot study was to verify the specificity in a number of the commercially available SARS-CoV-2 serological tests, using a panel of samples from patients with different chronic inflammatory diseases collected before the SARS-CoV-2 outbreak as negative controls, to get an indication of the extent of the issue for further developments, validations and verifications of serology assays.

## Materials and Methods

### Patient Serum Samples

To evaluate specificity of SARS-CoV-2 serological assays in patients with chronic inflammatory diseases, a selection of negative control samples was retrieved from the biobank (n=68). To exclude individuals with risk of previous exposure to SARS-CoV-2 infection, only samples collected before April 2019, (1-22 years since time of collection, **Table 1**), were included in the study. Serum samples were selected from patients with MS (n=10), RA (n=47), of which 2 samples were from the same patient), or SLE (n=10) (**Table 1**).

**Table 1:** Patients’ characteristics.

	Rheumatoid Arthritis	Multiple Sclerosis	Systemic Lupus Erythematosus
**Patients (n)**	47	10	10
**Age (years median, min - max)**	53 (18-71)	46 (39-70)	35.5 (30-60)
**Female (n, %)**	33 (70)	7 (70)	9 (90)
**IgM RF positive (n, %)**	24 (51)	n/a	0
**Anti-CCP2 positive** **(n, %)**	31 (66)	n/a	n/a
**Treated with IFX** **(n, %)**	7 (15)	0	0
**Treated with RTX** **(n, %)**	0	0	10 (100)
**Treated with IFN beta-1a (n, %)**	0	10 (100)	0
**ADA positive (n, %)**	n/a	3 (30)	5 (50)
**Time period of sampling**	1998 - 2006*	2003 - March 2019	2003 - 2018

n, number; RF, rheumatoid factor; anti-CCP, anti-cyclic citrullinated peptide; IFX, infliximab; RTX, rituximab; IFN interferon; ADA, anti-drug antibodies; n/a, not applicable; RA, rheumatoid arthritis. *Time period of sampling for RA patients treated with IFX: 2018 - March 2019.

The MS patients were diagnosed according to the 2017 updated McDonald criteria (19). The RA diagnoses were determined according to the 1987 revised American College of Rheumatology disease classification criteria by rheumatologists, within 12 months after the first symptoms of joint disease (20). For the SLE patients, the diagnoses were determined according to the American College of Rheumatism (21) and/or The Systemic Lupus Erythematosus Collaborating Clinics (22).

MS patient samples were collected in a research laboratory providing routine testing for anti-drug antibodies (ADAs) at the Centre for Molecular Medicine, Karolinska Institutet in Stockholm and had been treated with interferon beta (IFNβ). Three MS samples were ADA positive. Of the RA samples, 40 were from the Swedish population-based case control study Epidemiological Investigation of RA (EIRA) and had not been treated with any disease modifying anti-rheumatic drug (DMARD) (23). Of these patients, 20 were RF and anti-CCP2 positive (50%); six were RF negative but anti-CCP2 positive (15%), and 14 were both RF and anti-CCP2 negative (35%) (24). The additional seven RA patient samples were retrieved from a prospective study cohort (Sahlgrenska University Hospital, Gothenburg) and were infliximab (IFX) treated. Of these seven patient samples, three were RF and anti-CCP2 positive; two were RF negative but anti-CCP2 positive; one was RF positive but anti-CCP2 negative, and one sample was both RF and anti-CCP2 negative. The SLE samples were obtained from a study investigating the development of ADA against rituximab (RTX), and therefore all patients were RTX treated. Five of ten samples was anti-rituximab ADA positive (**Table S1**). All of the SLE patients had ANA and four of them anti-dsDNA.

This retrospective cohort study was approved by the Ethics Review Authority in Stockholm and Gothenburg (2020–23/04, dnr 2020-01649, 2012/1550-31/3, dnr 96-174). Samples and data were collected with informed consent in compliance with the Helsinki Declaration.

### Assays

#### Rheumatoid Factor Detection Methods

Analysis IgA, IgG and IgM isotypes of RA samles from the EIRA cohort and SLE samples was performed using the EliA immunoassay on the Phadia 2500 instrument and the cutoff values as stated in the manufacturer’s instructions (Phadia GmbH, Uppsala, Sweden) (24). Serum samples of RA patients treated with IFX were analyzed for IgM RF using laser nephelometry technique.

#### Anti-Cyclic Citrullinated Peptide Assay

Anti-CCP2 IgG in EIRA was previously determined using the Immunoscan CCPlus^®^ ELISA (Euro-Diagnostica, Malmö, Sweden), in accordance with the manufacturer´s instructions.

#### SARS-CoV-2 Serological Detection Methods

A total of 19 commercially available serological assays were evaluated in this study and compared to an in-house assay. Two Enzyme-Linked Immunosorbent Assays (ELISA) and 17 rapid diagnostic lateral flow assays (LFA) were included. These tests were assigned a letter from A – S (**Table 2**) and referred to as such in text and figures in this study. The brand name, antigen, manufacturer determined specificity and sensitivity, are outlined in **Table 2**. All tests were performed according to manufacturer instructions and using serum.

**Table 2:** Description of the SARS-CoV-2 serological assays and the test codes used in this study.

Test Code	Manufacturer	Kit Name	Antigen/Target	Catalogue number	Company reported assay specificity
**A**	Mikrogen Diagnostik GmbH, Germany*	recomWell SARS-CoV-2 IgG ELISA kit	Nucleocapsid protein	7304	IgG: 98.7%
**B**	Epitope Diagnostics, Inc., San Diego, USA	EDI Novel Coronavirus COVID-19 IgG ELISA Kit	Nucleocapsid protein	KT-1032	IgG: 100%
**C**	Jiangsu Medomics medical technology Co., Ltd, China	Rapid IgM-IgG combined Antibody Test Kit for SARS-CoV-2 (ICA)	Spike protein (RBD MK201027)	201030	Not specified
**D**	Salafa Oy, Salo, Finland	Salacor (Biohit) SARS-CoV-2 IgG/IgM rapid test kit	Nucleocapsid protein	COV-01-S	IgM: 99.2% IgG: 99.9%
**E**	Salafa Oy, Salo, Finland	Sienna SARS-CoV-2 IgG/IgM rapid test kit	Spike protein (RBD)	102222	IgM: 100% IgG: 98.8%
**F**	Liming Bio-Products Co., Ltd. Jiangsu, China	StrongStep_SARS-CoV-2 IgM/IgG_REF502090_ Antibody Rapid Test	Nucleocapsid and Spike protein	502090	IgM: 100% IgG: 98,7%
**G**	Zhejiang Orient Gene Biotech Co., Ltd. (China)	COVID-19 IgG/IgM Rapid Test Cassette alt. HEALGEN_ COVID-19 IgG/IgM Rapid Test Cassette (Whole Blood/Serum/Plasma_REF GCCOV-402a	Nucleocapsid and Spike protein (25)^	GCCOV-402a	IgM: 98.46% IgG: 98.46%
**H**	InTec Products inc., Haicang Xiamen, China	INTEC_ Colloidal Gold (whole blood/Serum/Plasma) Rapid SARS-CoV-2 Antibody (IgM/IgG)	Nucleocapsid protein (25)	ITP16001-TC25	Combined IgM+IgG: 98%
**I**	Sugentech Inc., South Korea*	SGTi-flex COVID-19 IgM/IgG	Nucleocapsid and Spike protein^	COVT025E	IgM: 98.3% (90% FDA August 2020) IgG:100%
**J**	Xiamen Biotime Biotechnology Co., Ltd. China	SARS-CoV2 IgG/IgM Rapid Qualitative Test	Spike protein^	BT1301	Not specified
**K**	Zhuhai Livzon Diagnostics Inc. (China)	COVID-19 IgG/IgM Lateral flow Rapid Test Cassette	Nucleocapsid protein	Not specified	IgM: 99.7% IgG: 99.4%
**L**	Abbott Point of Care Inc. USA*	Panbio COVID-19 lgG/lgM Rapid Test Device	Nucleocapsid protein (26)	ICO -T402	IgM: 92.8% IgG: 92.8%
**M**	SureScreen Diagnostics Ltd, UK	SureScreen Diagnostics COVID-19 IgG/IgM Rapid Test Cassette (Whole blood/serum/Plasma)	Spike protein/RBD	COVID19C	IgM: 99.2% IgG: 99.2%
**N**	Wuhan Easy Diagnosis Biomedicine Co., Ltd (China)*	COVID-19 (SARS-CoV-2) IgM/IgG Antibody Test kit	Not specified	SA-2-D	IgM: 100% IgG: 100%
**O**	Zhuhai Encode Medical Engineering Co., Ltd., China*	SARS-CoV-2 IgG/IgM Rapid test	S1-RBD and nucleocapsid protein	RCD-422	IgM: 100% IgG: 100%
**P**	Jiangsu SuperbioBiomedical Co., Ltd, China	SARS-CoV-2 (COVID-19) IgM/IgG Antibody Fast Detection Kit (Colloidal Gold)	Spike and nucleocapsid protein^	B00502	IgG: 95.8% IgM: 95.8%
**Q**	Lumigenex (Suzhou) Co., Ltd. China	Lumigenex SARS-CoV-2 IgG/IgM Antibody Rapid Test Kit	Spike and nucleocapsid protein	Not specified	Not specified
**R**	Wondfo, Guangzhou, China	Wondfo Biotech SARS-CoV-2 Antibody Test	Not specified	W195	Combined: IgM+IgG:99.57%
**S**	Innovita (Tangshan) Biological Technology Co Ltd China*	2019-nCoV Ab Test (Colloidal Gold)	Spike and Nucleocapsid protein^	Not specified	IgM: 100% IgG: 100%

^Emergency Use Authorization (EUA) Serology Test Performance | Food and Drug Administration (FDA).

*Stated in the instructions to have tested interference with RA and/or RF or other autoantibodies.

As of January 2021 tests C, G, I, K, N, O, P are not FDA approved.

For updated status of assays visit https://www.finddx.org/covid-19/pipeline.

#### Commercial Lateral Flow SARS-CoV-2 Assays

LFAs are designed to enable point of care analyses and can generate immediate results with read-outs as bands in small cartridges. These rapid lateral flow tests are developed for whole blood, serum and plasma. At time of testing, the appropriate volume of serum was applied to the designated well and then the buffer was added. After the recommended incubation period, the presence and intensity of the bands were investigated and graded from negative to four levels of positivity by the same operator.

#### An In-House Validated SARS-CoV-2 Serological Assay

The results were compared to an in-house multiplex bead-based and validated SARS-CoV-2 serological assay developed at SciLifeLab and KTH Royal Institute of Technology as previously described (27). In brief, IgG reactivity was analyzed in a high-throughput and multiplex bead-based format utilizing 384-well plates and FlexMap3D instrumentations (Luminex Corp) for read-out (27). Reactivity against three different in-house produced viral protein variants was used to differentiate between positive and negative samples: Spike trimers comprising the prefusion-stabilized spike glycoprotein ectodomain (28) (expressed in HEK and purified using a C-terminal Strep II tag), Spike S1 subunit (expressed in CHO and purified with HPC4 tag), and the Nucleocapsid protein (expressed in E. coli and purified using an N-terminal His-tag). The antigens were immobilized on magnetic color coded beads (MagPlex, Luminex Corp) and plasma/serum IgG that bound to the antigens were detected by an R-phycoerythrin conjugated goat anti-hIgG (Invitrogen, H10104). Reactivity against at least two out of the three viral antigens included in the panel was required for positive read out. The cut-off for seropositivity was defined as signals above the mean +6 SD of the 12 negative controls included in each assay. The method utilizing the combination of the three antigens has been found to have 99.2% sensitivity (99.6%, 99.2%, 96.7%, respectively, for the three antigens individually) and 99.8% specificity (98.9%, 99.1%, 98.4%, respectively, for the three antigens individually) based on 243 positive controls (defined as >16 days after onset or positive PCR) and 442 negative controls (defined as collected 2019 and earlier) samples.

##### Commercially Available SARS-CoV-2 ELISA Kits

The two included ELISAs were performed according to the manufacturers’ instructions. The first ELISA used to detect IgG against SARS-CoV-2 (test A, **Table 2**) was the recomWell SARS-CoV-2 IgG Elisa kit (Mikrogen Diagnostik GmbH, Germany). This assay is an indirect ELISA which uses highly purified recombinant nucleocapsid protein from SARS-CoV-2 as an antigen. The manufacturer had determined the potential interference of antibodies against other pathogens that might induce clinical symptoms similar to those of a SARS-CoV-2 infection (including for example seasonal coronaviruses, influenza A virus, RSV, *Mycoplasma pneumoniae*, *Chlamydia pneumoniae*). In addition, they also tested specificity using samples from people with conditions that present with atypical immune system activity including EBV infection, pregnancy, ANA and RF-positive subjects. The cut-off for positivity was calculated according to the manufacturer’s instructions.

The second ELISA test was the EDI Novel Coronavirus COVID-19 IgG Elisa Kit (Epitope Diagnostics, Inc., San Diego, USA) to detect IgG (test B, **Table 2**). This is an in vitro diagnostic and CE marked indirect ELISA with plates coated with peptides from the SARS-CoV-2 nucleocapsid antigen. Specificity of this assay was determined by the manufacturer using anti-influenza A, anti-influenza B, Hepatitis C virus (HCV), ANA and respiratory syncytial virus (RSV). The cut-off for positivity was determined according to the manufacturer’s instructions. The manufacturer states that a positive result may be due to past or present infection with SARS-CoV-2 but not due to other coronavirus strains, such as coronavirus HKU1, NL63, OC43, or 229E.

### Statistical Analyses

Rate of false positive signals were determined as the number of positive samples divided by the total number of samples tested for each assay. Statistical analyses and figures were generated using GraphPad Prism (version 8.2.1). The statistical difference between RF positive and RF negative RA subsets were calculated with Fishers exact test. The other groups were too small to make any meaningful statistical evaluations and thus these results are only presented as descriptive analyses.

## Results

### Commercial LFA and ELISA Assays

**Figure 1:**
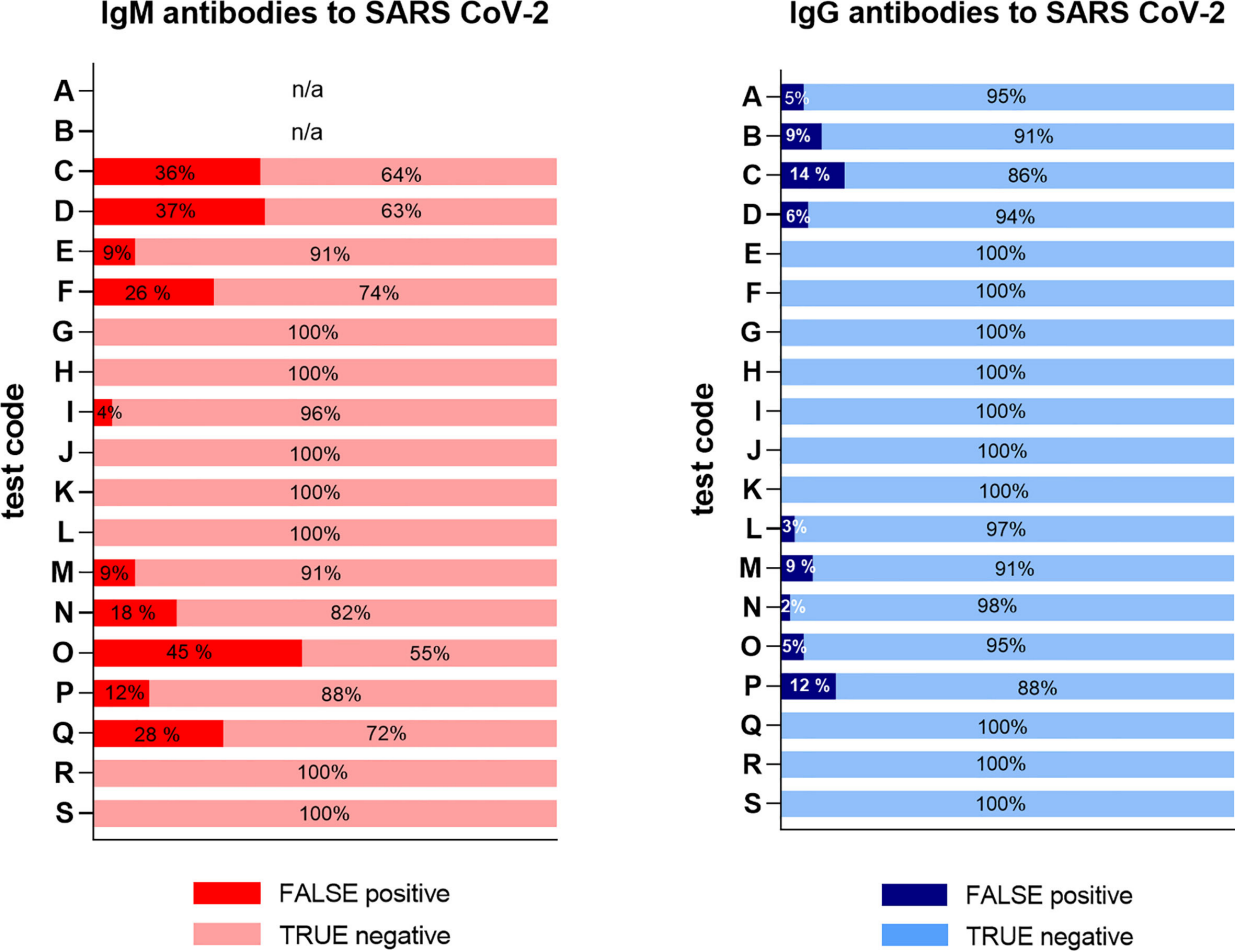
Overview of false positive results in all samples for 19 different serological tests. Six LFA tests (G, H, J, K, R & S) gave no false positive results. The false positivity rate of the remaining tests ranged between 2 - 45%. The test code keys are described in **Table 2**. The two ELISA assays (test A and B) were only tested for IgG.

Serum samples from 47 RA patients (with two samples from one of the patients), 10 SLE and 10 MS patients were evaluated using 19 SARS-CoV-2 commercial serological assays and compared to an in-house developed multiplex bead-based assay (27). The overall results of all 68 samples are illustrated in **Figure 1**. A total of six commercial LFAs (test G, H, J, K, R and S) reached 100% specificity for both IgG and IgM including all chronic inflammatory disease cohorts’ patients (*n*=67). Notably, all samples from MS patients (n=10) were negative for both IgM and IgG in all 20 assays.

**Figure 2:**
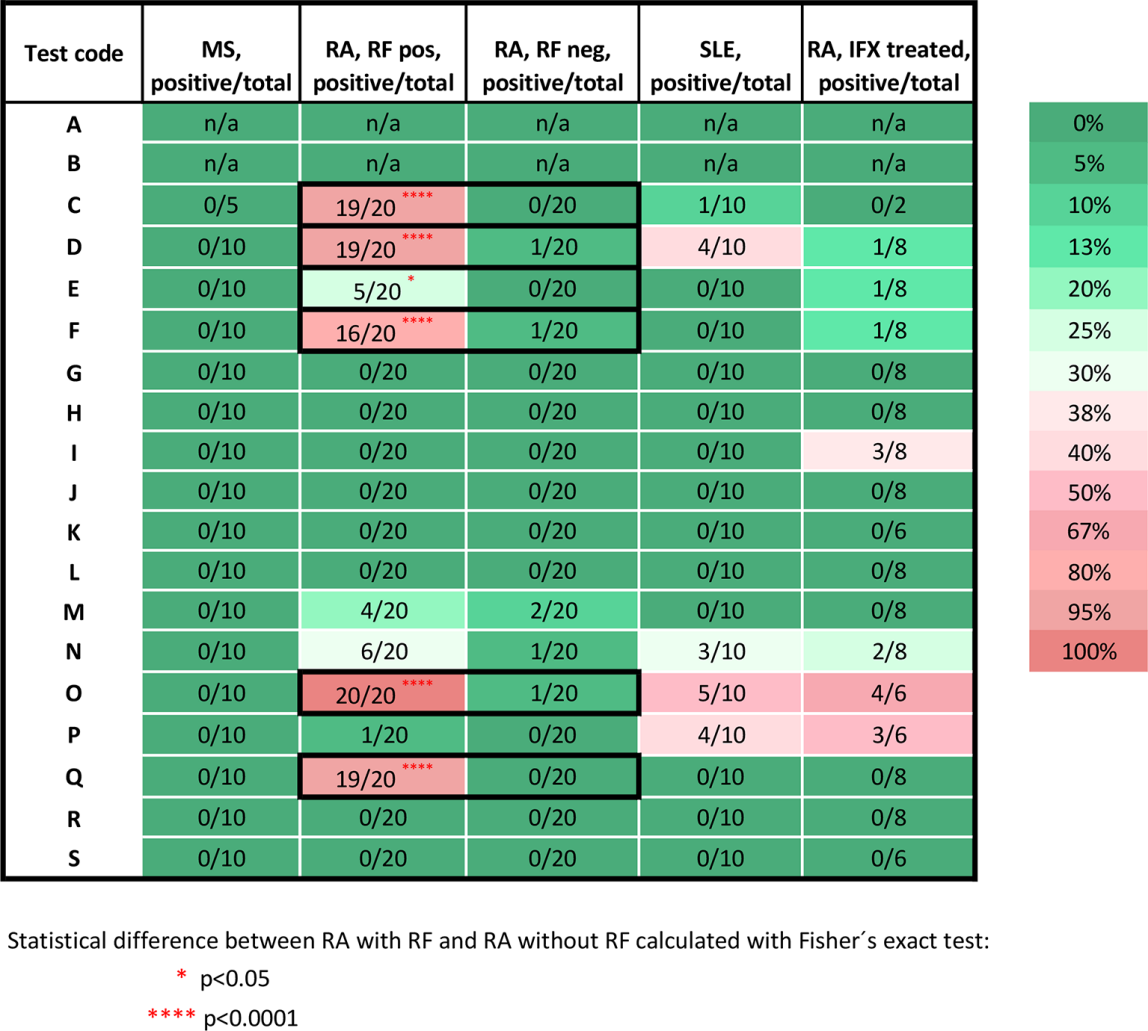
Percentage of the false positive test results for IgM antibodies against SARS-CoV-2. Samples from MS patients (n=10), DMARD naïve RF positive RA patients (n= 20), RF negative (n=20) RA patients, SLE patients (n=10), infliximab treated RA patients (n=8). The test code keys are described in **Table 2**. Stars indicate significant difference between RF status in RA patients, using Fisher exact test, *p < 0.05, ****p < 0.0001. Pos, positive; neg, negative; n, number; RF, rheumatoid factor; IFX, infliximab; n/a, not applicable; RA, rheumatoid arthritis; MS, multiple sclerosis; SLE, systemic lupus erythematosus.

**Figure 3:**
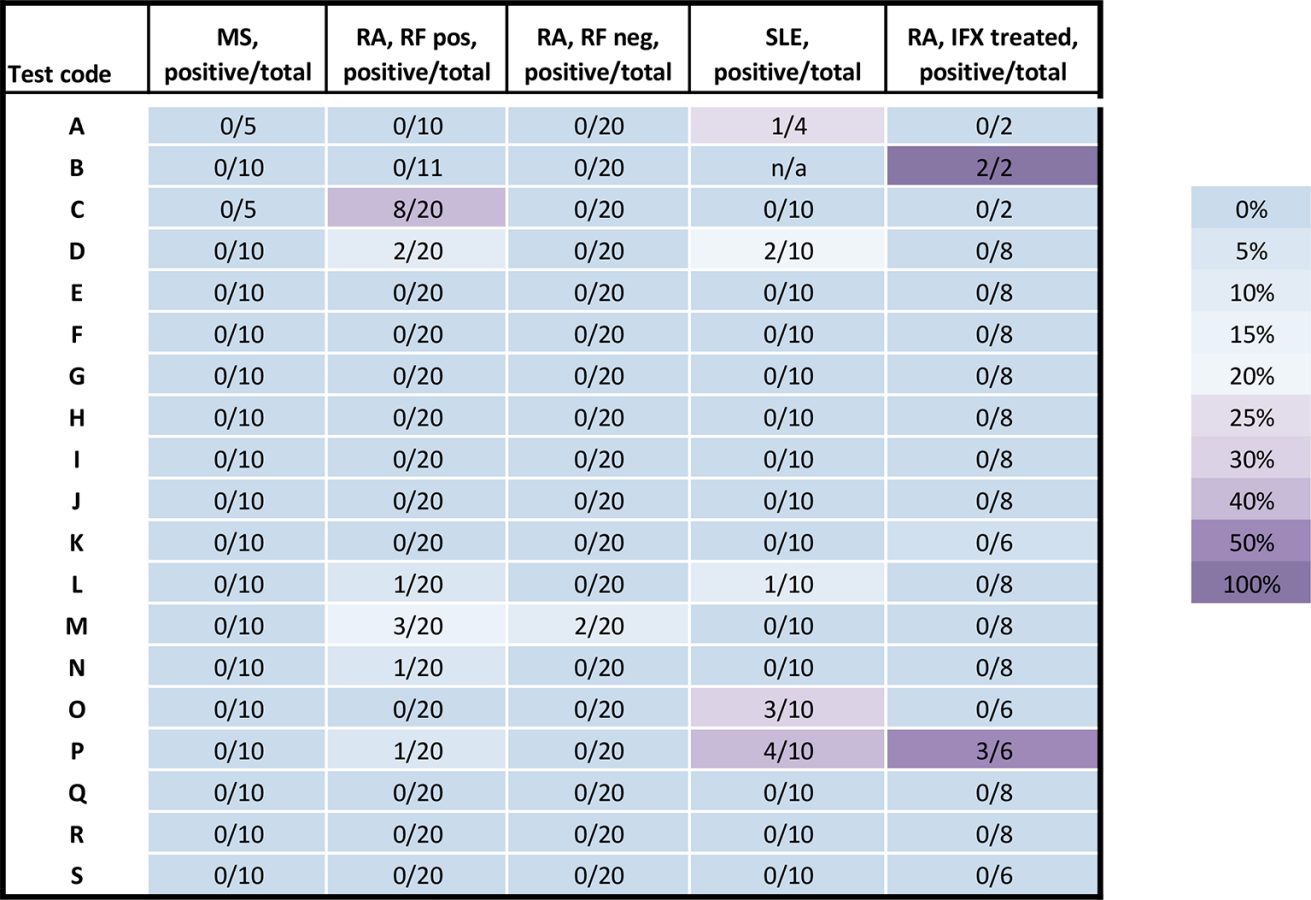
Percentage of the false positive test results for IgG. Samples from MS patients (n=10), DMARD naïve RF positive RA patients (n= 20), RF negative (n=20) RA patients, SLE patients (n=10), infliximab treated RA patients (n=8). The test code keys are described in **Table 2**. Pos, positive; neg, negative; n, number; RF, rheumatoid factor; IFX, infliximab; n/a, not applicable; RA, rheumatoid arthritis; MS, multiple sclerosis; SLE, systemic lupus erythematosus.

For the 17 LFAs evaluated for specificity using 25 RA samples (from 24 patients of which 20 were treatment naïve and 4 were treated with infliximab) that were positive for RF, 10 assays had unspecific signal detected for at least one immunoglobulin isotype (**Figures 2** and **3**). Five assays had unspecific signal for both IgM and IgG in a few up to a majority of the RA samples (test C: IgM 19/20, IgG 8/20; test D: IgM 19/20, IgG 2/20; test M: IgM 4/20, IgG 3/20; test N: IgM 6/20, IgG 1/20; and test P: IgM 1/20, IgG 1/20). Unspecific IgM signal, without unspecific IgG signal, was detected in four LFAs (test E: 5/20; test F: 16/20; test O: 20/20; and test Q: 19/20). In one LFA, only the IgG test gave unspecific signal (test L: 1/20). In contrast, only five assays detected unspecific signal in RA samples that were RF negative (*n*= 23), with five detecting IgM and one detecting IgG (test D: IgM 1/23; test F: IgM 1/23; test M: IgM 2/23, IgG 2/23; for test N: IgM 1/23; for test O: IgM 1/23) (**Figures 2** and **3**, **Table S1** for details). None of the two ELISAs (test A and B) gave any false positive signals with these samples. Due to insufficient sample volume, these ELISA tests could not be verified as extensively as the other tests (**Table S1** for details).

When using IFX treated-RA patients as SARS-CoV-2 serology negative controls (patients *n*=7, samples *n*=8) (**Figure 2**), unspecific signal was detected for IgM in seven assays (test D: 1/8; test E: 1/8; test F:1/8 test I: 3/8; test N: 2/8; test O: 4/6; and test P: 3/6) and for IgG (**Figure 3**) in two assays (test B: borderline positive signal in 2/2 samples and test P: 3 of 6 samples). Two samples were from one individual at two time points; prior to second infliximab infusion and after 9 months on treatment initiation. These two samples (IFX1 and IFX2 in **Table S1**) were both borderline positive in test B, and the sample taken after 9 months (IFX1) was positive in test E for IgM only.

False positive signal were detected in one ELISA test for IgG, five IgM LFAs and four IgG LFAs using the 10 SLE samples. All of these were ANA positive and four were anti-dsDNA positive (**Table S1**). Three of the SLE patients were IgA RF positive and one IgG RF positive. Five SLE patients had anti-rituximab ADA. There was no obvious pattern of associations to any of these antibodies with false positive signal in the SARS-CoV-2 serology assays.

### Serology Assay Specificities in Relation to Occurrence of RF Isotypes

The levels of IgG, IgM and IgA RF were very high in the RF positive RA samples (n=20), as these had been selected as such. Thus, associations between specific RF isotypes and false positive IgM/IgG anti-SARS-CoV-2 response could not be analyzed. Some indications could be retrieved from the SLE samples (n=10) who had a diversity of RF isotypes, i.e. none of the patients had IgM RF, three had IgA RF and one was positive for IgG RF. No associations were identified between RF isotypes and false positive anti-SARS-CoV-2 IgM or IgG signal in the SLE samples. However, there was a higher level of IgA RF and absence of false positive IgM/IgG anti-SARS-CoV-2 in two samples (SLE2 with 551 IU/ml and SLE7 with 26 IU/ml respectively in **Table S1**). These two samples were negative in all SARS-CoV-2 tests. Another two samples (SLE1 and SLE8 in **Table S1**) were negative for IgA RF but gave the highest number of false positive signals in SARS-CoV-2 tests. We also found that one RF negative SLE sample was IgM positive in two tests (C and N) and another RF negative SLE sample was both IgM and IgG positive in two tests (O and P). No associations were identified between anti-CCP2 antibodies or C1q-binding immune complexes and false positive IgM/IgG anti-SARS-CoV-2 response.

### SciLifeLab and KTH In-House Validated SARS-CoV-2 Serological Assay

Due to insufficient sample volume only 66 of the 68 samples were analyzed using the in-house developed multiplex bead-based assay for IgG detection as described above (27). All samples analyzed using this method were classified as negative. The only two samples not included were the two infliximab treated samples from the same patient (**Table S1**).

## Discussion

Serological assays are necessary tools in a pandemic, both for determining the proportion of the population already subjected to the infection and for the individual to confirm past infection and present immunity. In the case of SARS-CoV-2, it seems that a small proportion of the individuals who have been infected do not develop antibodies, at least not as determined by currently available serological assays (29). It also appears that some might have pre-existing immunity present in the population, as determined by memory T cell reactivity (30, 31) and the estimated prevalence of infected individuals in comparisons to the proportion that succumb in severe disease (32).

To elucidate these issues, we have to rely on the serological assays. Therefore, an independent verification of sensitivity and specificity of such assays is often required. These two are interconnected and a higher sensitivity often results in a lower specificity and vice versa. The diagnostic specificity of an assay is defined as the ability to correctly assign negative samples as negative. It can be determined by using a selection of samples negative for the new infection and positive for a range of other infections which might give cross-reactivity in the assay. Typically, the negativity for this pandemic can be guaranteed by having samples collected before SARS-CoV-2 emerged. When serological assays against viral antigen are developed, one major concern is regarding the cross-reactivity against similar viruses (16). SARS-CoV-2 serological assays using antigens that cross-react with antibodies generated towards other coronaviruses will not be approved, since the lower specificity would not serve the purpose of answering the clinically and epidemiologically important questions of who has developed antibodies against the new virus.

The aspect of immunity against SARS-CoV-2 is of particular importance to persons with chronic inflammatory diseases, given the concerns that treatments or their underlying disease might render them less able to fight the infection, establish immunity or respond to vaccinations. It is possible that only a few viral serology assays on the market will have tested for interferences using serum from patients with chronic inflammatory diseases. This may be because the priority is to ensure the assay as virus-specific in the sense of not cross-reacting with other viral infections. Alternatively, this may be because these types of patient sera are not easily accessible for manufacturers to be tested.

However, sera from patients with autoimmune diseases are notorious for interfering in immunological assays, giving higher background and unspecific signals. For instance, in the drug immunogenicity field, when validating assays for determining ADA, it is recommended to account for such unspecific signal during assay development and validation. This is achieved using a cohort of baseline samples in clinical trials from the targeted patient population who are treatment naive to the biological drug for which the ADA assay is developed (17, 18). For serological assays used to detect viral infections, such interference might not be discovered until more extensive screening of larger populations is started. This would become particularly notable, and give a false impression of exposure and immunity, if there is an interference by serum factors from patients with common diseases that have a frequency in the same magnitude as the studied infection. These serum factors could include autoantibodies, biological drugs, ADA, or aggregates and immune complexes formed e.g. by one or several of these components together. To complicate the matter further, there are indications that SARS-CoV-2 infection and COVID-19 disease might trigger these autoantibodies (33).

In the present study, a selection of samples from patients with chronic inflammatory diseases was used to determine the molecular specificity of a range of SARS-CoV-2 serological assays. We found that false positive results occur in the majority of the serological assays evaluated (**Figure 1**). Most notably, samples from RA patients with high levels of RF of IgM and IgG isotype resulted in a false positive signal in several assays (**Figures 2** and **3**). As RF binds to the constant parts of IgG, this could precipitate other antibodies present in an immunoassay in an unspecific way. These unspecific positive signals might not only give false indication of protective immunity to SARS-CoV-2 for an individual with RF but might also give an incorrect picture of the proportion of the population exposed to the infection during larger screenings, especially if the diagnoses or RF status of the population from where samples were collected are unknown. Most of the false positive signals were detected in the IgM assays, as has been noted by others (16, 34), which might be in line with the broader low affinity quality of the IgM antibodies, as compared to IgG class switched and affinity matured antibodies. Other studies have reported about this issue with different interpretations. One study using only one test (Innovita Biotechnology Co, Tangshan, China) reported that there was no interference with serum from persons with autoimmune disease (35), which we can confirm here for the Innovita LFA (test S).

Serum from patients with SLE has a high abundance of autoantibodies, including RF, ANA and antibodies against dsDNA. However, many other targets have also been described and the isotypes and specificities of these autoantibodies correlates with the symptoms of the disease (36). Although SLE is a less prevalent disease than RA, serum samples from these patients contributed essentially to the false positive signals in the present study.

Rheumatoid factor was the first autoantibody described in RA. According to different studies, RF has limited specificity for RA (from 48% to 92%) (37), since it can also be present in healthy controls and patients with other autoimmune and non-autoimmune diseases, such as chronic infections and cancer, and now also in COVID-19 survivors (33, 37–40). Since RF is heterophilic and can involve different immunoglobulin classes (IgM, IgG and IgA), we characterized these further. IgM-RF is the isotype commonly measured in most clinical laboratories, and detected in 60-80% of RA patients (37, 40), but might appear also in other diseases (37, 40, 41). In the current study, we were not able to evaluate any specific associations between occurrence of RF IgM or RF IgG and false positivity for IgM/IgG anti-SARS-Cov-2 in RA patients, since the RF positive RA sera were specifically selected to be highly positive for all RF isotypes simultaneously. Regarding the SLE samples, no associations were found between specific RF isotype and false positive signal in the SLE cohort. However, two cases of RF positive SLE patients were negative in all SARS-CoV-2 IgM and IgG antibody tests analyzed, indicating that the IgA isotype of the RF may not be an issue. The false positive signals in SLE samples observed in the present study might be explained by other autoantibodies such as ANA, anti-Sm/RNP, anti-Ro/La, anti-dsDNA. The exact biochemical interactions with RF in the SARS-CoV-2 serological assays need to be investigated further.

It could be argued that the unspecific signals detected in this study might actually be due to some underlying immunity, if there are mechanisms such as molecular mimicry behind the triggering of autoimmunity (42, 43) and these would also, hypothetically, work in the reverse direction. However, a more plausible explanation is that it is due to a technical difficulty in the assay development and thus one should not assume that these signals confirm any immunity against infection.

It should also be noted that samples used to determine the specificity of SARS-CoV-2 serological assays will be highly variable between manufacturers and often not reported in detail in the assay labels or information inserts. However, some manufacturers and vendors are aware of the issue and have included this in the information about the assay. Encode (test O) for example, report that specimen containing higher titers of heterophobic antibodies or RF may affect the results. With the RF positive RA samples used in the present study, 100% reacted in the IgM assay and 5% in the IgG assay, but also both RF positive and RF negative SLE samples gave signal. The Easy Diagnosis (test N) have tested RF, human anti mouse antibody (HAMA), and ANA and claim that they do not interfere with the kit, but in the present study we show that 5-30% of the samples gave a false positive signal. Innovita (test S) reported that samples positive for RF, ANA, HAMA have been analyzed, and they do not report cross reactivity in their test, as could also be independently verified by us.

Mikrogen Diagnostik (test A) reported cross-reactivity with RF, but we could only identify cross-reactivity in the IgG assay with an RF negative SLE sample. Abbott (test L) reported that they tested samples positive for RF (3/3), HAMA (3/3), and ANA (3/3), and that these did not affect the performance of the test. However, we see two false positive signals in the IgG test for RF positive RA and SLE samples. Sugentech (test I) reported no cross-reactivity with anti-human IgG, IgM, IgA and IgE, but here we report false positive signal in three samples from an IFX treated patients. Notably, for Sienna (test E), two samples from an IFX treated patient were tested, one sample taken before (IFX2, **Table S1**) and one sample taken after IFX treatment (IFX1, **Table S1**). Only the sample taken while the patients were on treatment gave false positive signal in the IgM test. This rises a possible additional concern regarding factors that can interfere in serology assays, given how many people who currently are treated with infliximab. However, since no other IFX treated RA sample reacted, it argues against it. The IFX treated RA sample also had high ADA, which then potentially could be the interfering factor. These findings have to be verified in a larger cohort. The Sienna (test E) also gave false positive signal for five of the RF positive untreated RA samples and since the IFX treated RA also was positive for RF, this might have been the interfering factor.

It should be noted that several serology tests did not give any false positive results with these complicated sera and thus there are methods to avoid unwanted interference. A suggested method to resolve the RF issue, at least in ELISA tests, is to use urea for dissociation of the interfering signals (44).

There are some limitations in this study. Firstly, there are over 400 SARS-CoV-2 serology assays available on the market and with the limited set of stored samples, we could only analyze a fraction of these. For reliable use of serological assays for patients with chronic inflammatory diseases, each assay would need to be individually analyzed with SARS-CoV-2 negative serum from that patient population, before one starts to screen that patient group. Here we can only report the specificity in relation to MS, RA and SLE. Due to the limited availability of sample material, only one test result per sample, per assay, was retrieved and it was not possible to further elucidate the molecular mechanism behind the positive signals. To delineate the molecular explanation of the false positive signal, several more factors would need to be known about the samples, including more extensive information on RF, biological treatments and ADA. Ideally, a well characterized common set of serum samples should be made available to verify several additional serological tests in parallel. Secondly, the LFA’s are primary made for whole blood, to enable individual to do a rapid test with a drop a blood from the fingertip, but here we only had stored serum to use for testing. However, all of the assays also indicate that they work with serum and plasma. Given that the serum from MS patients did not give any signal in any assay, the false positive signals detected in this study are most probably not an issue of having a different matrix, but more likely the unspecific antibody contents of the serum.

In conclusion, serological assays could be sensitive to interfering antibodies, as shown in sera from persons with autoimmune diseases. There is a trade-off between requiring extensive screening for unspecific binding in these assays and the harm of delaying the process of making these assays available for emergency use during a pandemic. A cost benefit analysis including all these aspects has to be made on both national and global level. However, if persons with autoimmune disease, health care providers and decision makers are aware about this issue, they could adapt the testing strategy and selecting kits only passing these specificity requirements. To enable such informed decisions, it would be helpful if information about which types and number of samples have been used for validation of specificity is clearly stated in the label of all tests.

## Data Availability Statement

The raw data supporting the conclusions of this manuscript will be made available by the authors, without undue reservation, to any qualified researcher.

## Ethics Statement

The studies involving human participants were reviewed and approved by the Ethics Review Authority in Stockholm and Gothenburg (2020–23/04, dnr 2020-01649, 2012/1550-31/3, dnr 96-174). Samples and data were collected with informed consent in compliance with the Helsinki Declaration. The ethics committee waived the requirement of written informed consent for participation.

## Author Contributions

All authors contributed to conceptualization, execution, writing, review, and editing of the manuscript. All authors approved the final version of the manuscript.

## Funding

This work was supported by grants from the Dr. Margaretha Nilsson’s Foundation for Medical Research, Vinnova (grant number 2020-02865) and the PCORI [Patient-Centered Outcomes Research Institute] contract MS-1511-33196 for Funded Research Project Standard CR8 with Karolinska Institutet for the project: “Rituximab in Multiple Sclerosis: A Comparative Study on Effectiveness, Safety, and Patient-Reported Outcomes”. Autoantibody analyses in EIRA was supported by grants from the Swedish Research Council, King Gustav V:s 80-year foundation, and the EU/EFPIA IMI project RTCure (grant n° 777357). Autoantibody analyses in Bio-ADA cohort were supported by the grants from Professor Nanna Svartz Foundation, the Gothenburg Medical Society (GLS-889421), and the Regional agreement on medical training, clinical research between the Western Götaland county council and the University of Gothenburg (ALFGBG-926621) and Aina (Ann) Wallströms och Mary-Ann Sjöbloms Foundation for Medical Research.

## Conflict of Interest

The authors declare that the research was conducted in the absence of any commercial or financial relationships that could be construed as a potential conflict of interest.

## References

[1] Bar-On YM Flamholz A Phillips R Milo R. Sars-CoV-2 (Covid-19) by the Numbers. Elife (2020) 9:1–15. 10.7554/eLife.57309 PMC7224694 32228860

[2] Dong E Du H Gardner L. An Interactive Web-Based Dashboard to Track COVID-19 in Real Time. Lancet Infect Dis (2020) 20(5):533–4. 10.1016/S1473-3099(20)30120-1 PMC7159018 32087114

[3] Luna G Alping P Burman J Fink K Fogdell-Hahn A Gunnarsson M. Infection Risks Among Patients With Multiple Sclerosis Treated With Fingolimod, Natalizumab, Rituximab, and Injectable Therapies. JAMA Neurol (2019) 77(2):184–91. 10.1001/jamaneurol.2019.3365 PMC6784753 31589278

[4] Loonstra FC Hoitsma E van Kempen ZL Killestein J Mostert JP. Covid-19 in Multiple Sclerosis: The Dutch Experience. Mult Scler (2020) 26(10):1256–60. 10.1177/1352458520942198 PMC7493197 32662742

[5] Sormani MP. Italian Study Group on C-iims. An Italian Programme for COVID-19 Infection in Multiple Sclerosis. Lancet Neurol (2020) 19(6):481–2. 10.1016/S1474-4422(20)30147-2 PMC7191287 32359409

[6] Quartuccio L Valent F Pasut E Tascini C De Vita S. Prevalence of COVID-19 Among Patients With Chronic Inflammatory Rheumatic Diseases Treated With Biologic Agents or Small Molecules: A Population-Based Study in the First Two Months of COVID-19 Outbreak in Italy. Joint Bone Spine (2020) 87(5):439–43. 10.1016/j.jbspin.2020.05.003 PMC7239017 32445935

[7] El-Gabalawy H Guenther LC Bernstein CN. Epidemiology of Immune-Mediated Inflammatory Diseases: Incidence, Prevalence, Natural History, and Comorbidities. J Rheumatol Suppl (2010) 85:2–10. 10.3899/jrheum.091461 20436161

[8] Collaborators GBDMS. Global, Regional, and National Burden of Multiple Sclerosis 1990-2016: A Systematic Analysis for the Global Burden of Disease Study 2016. Lancet Neurol (2019) 18(3):269–85. 10.1016/S1474-4422(18)30443-5 PMC6372756 30679040

[9] Cross M Smith E Hoy D Carmona L Wolfe F Vos T. The Global Burden of Rheumatoid Arthritis: Estimates From the Global Burden of Disease 2010 Study. Ann Rheum Dis (2014) 73(7):1316–22. 10.1136/annrheumdis-2013-204627 24550173

[10] Waller M Toone EC Vaughan E. Study of Rheumatoid Factor in a Normal Population. Arthritis Rheum (1964) 7:513–20. 10.1002/art.1780070507 14212945

[11] Jonsson T Thorsteinsson J Valdimarsson H. Does Smoking Stimulate Rheumatoid Factor Production in non-Rheumatic Individuals? APMIS (1998) 106(10):970–4. 10.1111/j.1699-0463.1998.tb00247.x 9833699

[12] Tsokos GC Lo MS Costa Reis P Sullivan KE. New Insights Into the Immunopathogenesis of Systemic Lupus Erythematosus. Nat Rev Rheumatol (2016) 12(12):716–30. 10.1038/nrrheum.2016.186 27872476

[13] Fedrigo A Dos Santos T Nisihara R Skare T. The Lupus Patient With Positive Rheumatoid Factor. Lupus (2018) 27(8):1368–73. 10.1177/0961203318759607 29460700

[14] Baumgarth N Nikolich-Zugich J Lee FE Bhattacharya D. Antibody Responses to SARS-CoV-2: Let’s Stick to Known Knowns. J Immunol (2020) 205(9):2342–50. 10.4049/jimmunol.2000839 PMC7578055 32887754

[15] Pallett SJC Rayment M Patel A Fitzgerald-Smith SAM Denny SJ Charani E. Point-of-Care Serological Assays for Delayed SARS-CoV-2 Case Identification Among Health-Care Workers in the UK: A Prospective Multicentre Cohort Study. Lancet Respir Med (2020) 8(9):885–94. 10.1016/S2213-2600(20)30315-5 PMC7380925 32717210

[16] Whitman JD Hiatt J Mowery CT Shy BR Yu R Yamamoto TN. Evaluation of SARS-CoV-2 Serology Assays Reveals a Range of Test Performance. Nat Biotechnol (2020) 38(10):1174–83. 10.1038/s41587-020-0659-0 PMC7740072 32855547

[17] Gupta S Devanarayan V Finco D Gunn GR3 Kirshner S Richards S. Recommendations for the Validation of Cell-Based Assays Used for the Detection of Neutralizing Antibody Immune Responses Elicited Against Biological Therapeutics. J Pharm BioMed Anal (2011) 55(5):878–88. 10.1016/j.jpba.2011.03.038 21531522

[18] Devanarayan V Smith WC Brunelle RL Seger ME Krug K Bowsher RR. Recommendations for Systematic Statistical Computation of Immunogenicity Cut Points. AAPS J (2017) 19(5):1487–98. 10.1208/s12248-017-0107-3 28733862

[19] Thompson AJ Banwell BL Barkhof F Carroll WM Coetzee T Comi G. Diagnosis of Multiple Sclerosis: 2017 Revisions of the McDonald Criteria. Lancet Neurol (2018) 17(2):162–73. 10.1016/S1474-4422(17)30470-2 29275977

[20] Arnett FC Edworthy SM Bloch DA McShane DJ Fries JF Cooper NS. The American Rheumatism Association 1987 Revised Criteria for the Classification of Rheumatoid Arthritis. Arthritis Rheum (1988) 31(3):315–24. 10.1002/art.1780310302 3358796

[21] Tan EM Cohen AS Fries JF Masi AT McShane DJ Rothfield NF. The 1982 Revised Criteria for the Classification of Systemic Lupus Erythematosus. Arthritis Rheum (1982) 25(11):1271–7. 10.1002/art.1780251101 7138600

[22] Petri M Orbai AM Alarcon GS Gordon C Merrill JT Fortin PR. Derivation and Validation of the Systemic Lupus International Collaborating Clinics Classification Criteria for Systemic Lupus Erythematosus. Arthritis Rheum (2012) 64(8):2677–86. 10.1002/art.34473 PMC3409311 22553077

[23] Stolt P Bengtsson C Nordmark B Lindblad S Lundberg I Klareskog L. Quantification of the Influence of Cigarette Smoking on Rheumatoid Arthritis: Results From a Population Based Case-Control Study, Using Incident Cases. Ann Rheum Dis (2003) 62(9):835–41. 10.1136/ard.62.9.835 PMC1754669 12922955

[24] Reed E Hedstrom AK Hansson M Mathsson-Alm L Brynedal B Saevarsdottir S. Presence of Autoantibodies in “Seronegative” Rheumatoid Arthritis Associates With Classical Risk Factors and High Disease Activity. Arthritis Res Ther (2020) 22(1):170. 10.1186/s13075-020-02191-2 32678001 PMC7364538

[25] GeurtsvanKessel CH Okba NMA Igloi Z Bogers S Embregts CWE Laksono BM. An Evaluation of COVID-19 Serological Assays Informs Future Diagnostics and Exposure Assessment. Nat Commun (2020) 11(1):3436. 10.1038/s41467-020-17317-y 32632160 PMC7338506

[26] Batra R Olivieri LG Rubin D Vallari A Pearce S Olivo A. A Comparative Evaluation Between the Abbott Panbio COVID-19 IgG/IgM Rapid Test Device and Abbott Architect SARS Cov-2 IgG Assay. J Clin Virol (2020) 132:104645. 10.1016/j.jcv.2020.104645 32961429 PMC7493757

[27] Rudberg AS Havervall S Manberg A Jernbom Falk A Aguilera K Ng H. Sars-CoV-2 Exposure, Symptoms and Seroprevalence in Healthcare Workers in Sweden. Nat Commun (2020) 11(1):5064. 10.1038/s41467-020-18848-0 33033249 PMC7544689

[28] Wrapp D Wang N Corbett KS Goldsmith JA Hsieh CL Abiona O. Cryo-EM Structure of the 2019-Ncov Spike in the Prefusion Conformation. Science (2020) 367(6483):1260–3. 10.1126/science.abb2507 PMC7164637 32075877

[29] Kellam P Barclay W. The Dynamics of Humoral Immune Responses Following SARS-CoV-2 Infection and the Potential for Reinfection. J Gen Virol (2020) 101(8):791–7. 10.1099/jgv.0.001439 PMC7641391 32430094

[30] Grifoni A Weiskopf D Ramirez SI Mateus J Dan JM Moderbacher CR. Targets of T Cell Responses to SARS-CoV-2 Coronavirus in Humans With COVID-19 Disease and Unexposed Individuals. Cell (2020) 181(7):1489–501 e15. 10.1016/j.cell.2020.05.015 32473127 PMC7237901

[31] Sekine T Perez-Potti A Rivera-Ballesteros O Stralin K Gorin JB Olsson A. Robust T Cell Immunity in Convalescent Individuals With Asymptomatic or Mild Covid-19. Cell (2020) 183(1):158–68.e14. 10.1016/j.cell.2020.08.017 32979941 PMC7427556

[32] Li R Pei S Chen B Song Y Zhang T Yang W. Substantial Undocumented Infection Facilitates the Rapid Dissemination of Novel Coronavirus (SARS-Cov-2). Science (2020) 368(6490):489–93. 10.1126/science.abb3221 PMC7164387 32179701

[33] Woodruff M Ramonell RP Lee FE-H Sanz I. Clinically Identifiable Autoreactivity is Common in Severe SARS-CoV-2 Infection. medRxiv (2020), 2020.10.21.20216192. 10.1101/2020.10.21.20216192

[34] Latiano A Tavano F Panza A Palmieri O Niro GA Andriulli N. False Positive Results Of IgM/IgG Antibodies Against Antigen of the SARS-CoV-2 in Sera Stored Before the 2020 Endemia in Italy. Int J Infect Dis (2020) 104:159–63. 10.1016/j.ijid.2020.12.067 PMC7834192 33383223

[35] Teng J Dai J Su Y Zhou Z Chi H Wan L. Detection of IgM and IgG Antibodies Against SARS-CoV-2 in Patients With Autoimmune Diseases. Lancet Rheumatol (2020) 2(7):e384–e5. 10.1016/S2665-9913(20)30128-4 PMC7234786 32835238

[36] Dema B Charles N. Autoantibodies in SLE: Specificities, Isotypes and Receptors. Antibodies (Basel) (2016) 5(1):2. 10.3390/antib5010002 PMC6698872 31557984

[37] Rocha SD Baldo DC Andrade LEC. Clinical and Pathophysiologic Relevance of Autoantibodies in Rheumatoid Arthritis. Adv Rheumatol (2019) 59:2. 10.1186/s42358-018-0042-8 30657101

[38] Verheul MK Fearon U Trouw LA Veale DJ. Biomarkers for Rheumatoid and Psoriatic Arthritis. Clin Immunol (2015) 161(1):2–10. 10.1016/j.clim.2015.04.005 25934385

[39] Ingegnoli F Castelli R Gualtierotti R. Rheumatoid Factors: Clinical Applications. Dis Markers (2013) 35(6):727–34. 10.1155/2013/726598 PMC3845430 24324289

[40] Nishimura K Sugiyama D Kogata Y Tsuji G Nakazawa T Kawano S. Meta-Analysis: Diagnostic Accuracy of Anti-Cyclic Citrullinated Peptide Antibody and Rheumatoid Factor for Rheumatoid Arthritis. Ann Intern Med (2007) 146(11):797–808. 10.7326/0003-4819-146-11-200706050-00008 17548411

[41] Jonsson T Steinsson K Jonsson H Geirsson AJ Thorsteinsson J Valdimarsson H. Combined Elevation of IgM and IgA Rheumatoid Factor has High Diagnostic Specificity for Rheumatoid Arthritis. Rheumatol Int (1998) 18(3):119–22. 10.1007/s002960050069 9833253

[42] Sospedra M Martin R. Molecular Mimicry in Multiple Sclerosis. Autoimmunity (2006) 39(1):3–8. 10.1080/08916930500484922 16455577

[43] Wucherpfennig KW Strominger JL. Molecular Mimicry in T Cell-Mediated Autoimmunity: Viral Peptides Activate Human T Cell Clones Specific for Myelin Basic Protein. Cell (1995) 80(5):695–705. 10.1016/0092-8674(95)90348-8 7534214 PMC7133435

[44] Wang Q Du Q Guo B Mu D Lu X Ma Q. A Method to Prevent SARS-Cov-2 IgM False Positives in Gold Immunochromatography and Enzyme-Linked Immunosorbent Assays. J Clin Microbiol (2020) 1–7:e00375–20. 10.1128/JCM.00375-20 PMC7269408 32277023

